# Grazing behavior of dairy cows under contrasting pasture allocation frequencies and areas

**DOI:** 10.3168/jdsc.2023-0478

**Published:** 2024-02-29

**Authors:** L.J. Farrell, C. Glassey, C. Burke, Y. Lopez Moreno, J.P. Edwards

**Affiliations:** 1 DairyNZ Ltd., Private Bag 3221, Hamilton, New Zealand 3240; 2 DairyNZ Ltd., PO Box 85066, Lincoln University, Lincoln, New Zealand 7647

## Abstract

This study aimed to compare grazing behavior of dairy cows with highly contrasting pasture allocation frequencies. The study ran from September 9, 2022, to December 2, 2022 (12 wk), during a time when daily pasture growth was expected to exceed daily herd intake. Three pasture allocation frequencies were compared, each with 11 spring-calving cows grazing a 4-ha farmlet. The allocations were high frequency rotational grazing (HFRG; 32.5 m^2^/cow per allocation and 4 allocations/d), rotational grazing with weekly allocations (7RG; 909 m^2^/cow per allocation and each allocated area continuously grazed over 7 d), and continuous grazing (CG; 1,818 m^2^/cow per allocation). Animal behavior was monitored using IceQube accelerometers (Peacock Technology) and CowManager ear tags (Agis Automatisering BV). Milk yield and composition were measured and pasture cover was estimated. Daily eating time was highest for CG animals, whereas lying time, lying bouts, and ruminating time were lowest, with no differences between HFRG and 7RG. There were no differences in fat- and protein-corrected milk yield between farmlets; however, estimated accumulated pasture yield was greater with more frequent allocations. Although milk production did not differ between treatment groups, this appeared to be achieved through greater eating times when allocated pasture less frequently, at the expense of time spent lying.

## Highlights

• With less frequent pasture allocation, eating time increased and lying time decreased.
• Lying time was lowest for continuously grazing (set stocked) cows.
• Behavior was different each day for cows with weekly pasture allocations.
• Milk solids production did not differ, but pasture growth was less with fewer allocations.

## Graphical Abstract

**Figure:**
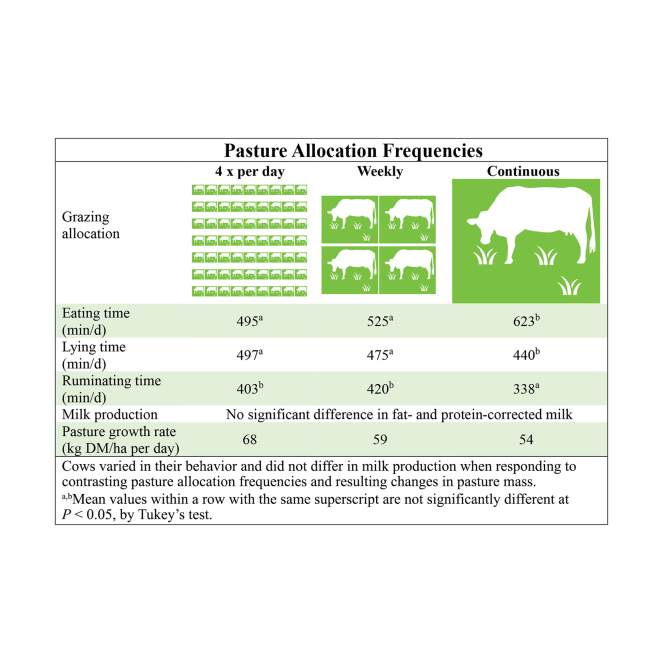
**Summary** Lowering demand for farm labor and decision-making by allocating cows to fresh pasture less frequently will reduce pasture growth but not milk production in the short term. Our results indicate that cows did not differ in fat- and protein-corrected milk production through spending more time eating when allocated pasture less frequently, at the expense of time spent lying.

Most pasture-based dairy farms practice rotational grazing, with cows normally allocated fresh pasture on a daily or twice daily basis, rather than continuous grazing (**CG**) where animals graze the same area over an extended period of time. Historic multiyear farmlet studies identified smaller effects on milk production relative to reductions in accumulated pasture with less frequent allocations, where stocking rate was unchanged (McMeekan, 1956; Lucas, 1958; McFeely et al., 1975), though one study found no impacts on either milk or pasture yields (Schelpers and Lantinga, 1985). While impacts of pasture allocation frequency on milk and pasture yields have been quantified, impacts on grazing behavior have received less attention and effects on lying behavior have not been reported to date. Longer daily eating time has been reported for continuously grazing cows compared with rotationally grazing cows (Pulido and Leaver, 2003). While no differences in daily eating time were observed for cows with pasture allocated daily or every 4 d, with daily grazing time increased during the 4-d allocation period (Abrahamse et al., 2008). As time is a limited resource, effects of pasture allocation frequency on daily eating time can be expected to influence time spent engaging in other behaviors such as lying and socializing (Løvendahl and Munksgaard, 2016). Lying time is important for cow welfare (Munksgaard and Simonsen, 1996), and understanding how management changes, such as changing pasture allocation frequencies, influence cows' time budgets is of interest.

There is interest in pasture allocation frequency due to current challenges in meeting labor requirements. Reduced workload and cognitive load for farmers via pasture and grazing management could be achieved through using less frequent pasture allocations, resulting in fewer grazing management decisions and batching of tasks such as postgrazing fertilizer applications or weed control over a larger area, though labor for herding may change with larger grazing areas unless technology such as virtual herding is used. We hypothesized that contrasting pasture allocation frequencies would affect cows' behavior and daily time budgets.

With approval from the AgResearch Animal Ethics Committee (application #479), this study ran from September 9 to December 2, 2022, at DairyNZ Scott Farm (Newstead, New Zealand; 37.77°S, 175.37°W). Thirty-three lactating dairy cows were blocked for age (mean 4.7 yr ± 2.3 SD), calving date (mean July 29, 2022 ± 6.5 d), and pretrial daily milk yield (means of 24.9 ± 4 kg or 2.1 ± 0.3 kg of fat + protein) and randomly allocated to 1 of 3 groups. Postallocation there were no significant differences between groups. Each herd was allocated 3 first-calving heifers (27% of group) and 4 ha of pasture (stocking rate of 2.75 cows/ha). Each group was assigned a pasture allocation frequency: high frequency rotational grazing (**HFRG**) where fresh pasture was allocated 4 times daily between 0700 and 1700 h, equivalent to 32.5 m^2^/cow per allocation; fresh pasture allocated weekly each Friday (**7RG**) equivalent to 909 m^2^/cow per allocation with each allocated area continuously grazed over 7 d; or CG with access to all 4 ha on a daily basis with 2 ha offered in the morning and the other 2 ha offered in the afternoon, equivalent to 1,818 m^2^/cow per allocation. Cows in the HFRG treatment were not back fenced, meaning they had access to 130 m^2^/cow per day. Both rotational grazing treatments were allocated an equivalent of 1/28th of their 4 ha each day, which resulted in a 21- and 28-d grazing interval for 7RG and HFRG, respectively. Each cow was fitted with a CowManager ear-tag (Agis Automatisering BV, Harmelen, the Netherlands), which recorded minutes per hour spent eating, ruminating, active, high active, and not active and an IceQube accelerometer (Peacock Technology, Stirling, Scotland), which recorded every 15 min the number of steps, number of transitions from lying to standing, and the total lying time. IceQubes were attached 2 d immediately preceding the experimental period while all cows grazed together with daily pasture allocations. During this time there were no significant (*P* > 0.05) differences in any of the metrics reported. Milk weights were recorded at each of the 2 daily milkings with weekly samples (Monday p.m. and Tuesday a.m.) collected for composition analysis. An additional milk sample was collected on Thursday p.m. and Friday a.m. for 7RG due to variation in pasture availability; results were averaged weekly. Cow liveweight and BCS were measured every 14 d.

Each 4 ha farmlet was managed to have a similar starting average pasture cover (**APC**) of 1,980 kg of DM/ha on September 9, a date when historically pasture growth is expected to equal and subsequently exceed feed demand at this location and stocking rate. The APC for all 3 farmlets was monitored weekly each Tuesday by calibrated visual assessment (Lile et al., 2001). Compressed pasture height was measured by rising plate meter pregrazing each Wednesday (HFRG) and Friday (7RG), and postgrazing each Friday (for both HFRG and 7RG). Total rainfall during the study period was 435 mm, higher than the 10-year average of 265 mm. With unexpected pasture deficits influenced by the climate, 1 cow was removed from the CG farmlet in wk 6 (when APC was <1,800 kg of DM/ha) as required in the animal ethics approval to maintain sufficient feed availability. A zero value for milk yield, liveweight, and BCS (and subsequent back-calculations using these values) was assigned to her after removal to ensure that group averages were comparable for the 4 ha farmlet area. A single bale of silage was fed to cows in the 7RG farmlet in wk 7 for the same reasons. Surplus pasture was conserved as baleage from the HFRG (in wk 8 and 12) and 7RG (in wk 12) farmlets. Pasture quality was monitored fortnightly from samples collected to grazing height (~40 mm) in the next areas to be offered (for HFRG and 7RG), and across the entire 4 ha farmlet for CG. Samples were bulked per treatment, well mixed, subsampled, dried at 60°C for 48 h and ground to 1 mm. Analysis for digestible organic matter in the dry matter (**DOMD**) used near-infrared spectrophotometry (MPA FT-NIR Analyzer, Bruker Optics, Billerica, MA) at Hill Laboratories (Hamilton, New Zealand). Metabolizable energy concentration (MJ/kg of DM) was estimated using the equation ME = DOMD × 0.16, and the quality of pasture baleage fed as supplement was analyzed using the same method.

Treatment effects for milk production and animal characteristics were investigated using a mixed models approach to repeated measures ANOVA. The model allowed for heterogeneous variance over time and between groups. The cow was the observational unit (n = 33) and the herd (n = 3) was the experimental unit, with repeated measures through time (n = 12 wk). Behavioral data (CowManager and IceQube) were summed by day (except lying bouts) and then averaged by week for average daily totals per cow and week. Milk and milk component yields for each milking were averaged by week, and p.m. and a.m. values summed to obtain average daily yields for each cow and week. Average daily fat- and protein-corrected milk (**FPCM**) yield was calculated using IDF (2016) equations. Daily ME requirements by cow and week were estimated using the formulas published by Nicol and Brookes (2007). The repeated measures models included treatment, week, and their interaction as fixed effects, pretreatment measurement as a covariate (except behavior), and cow or paddock as a random factor. The covariance pattern model chosen was autoregressive. For estimating effects of day of the week, daily values were averaged across week for each cow and day of week. The repeated measures model used included treatment, day of week, and their interaction as fixed effects, pretreatment measurement as a covariate (except behavior), and cow as a random factor. A compound symmetry covariance pattern model was chosen. Pre- and postgrazing heights were averaged by week and analyzed via one-way ANOVA using weeks as replicates. Analyses of variance were followed by pairwise comparisons between treatments within and across time points using Tukey adjustment for multiple comparisons. Results are presented as least squares means and standard difference of means, as well as *P*-values for the effect of treatment. Statistical analyses were performed using SAS 9.4 (SAS Institute Inc., Cary, NC) and significance was declared if *P* ≤ 0.05. Pasture growth rate for the CG treatment could not be determined through pasture measurement due to constant presence of grazing cows. Therefore, it was estimated using back-calculated energy requirements for milk production, the net amount of pasture baleage made and fed, the net change in APC between start and end dates of the experiment, and the pasture and baleage ME content (mean pasture ME across the experimental period was 11.7 MJ of ME/kg of DM). Baleage harvested, change in APC, and estimated pasture growth are presented on a per hectare basis and were not analyzed statistically.

Cows in the CG treatment had greater eating time, and less ruminating time, lying bouts, and lying time (all *P* < 0.05) than HFRG and 7RG cows, which were not different from each other (Table 1, Figure 1). The higher eating time and step counts for CG suggest these cows covered greater areas to achieve their daily pasture intakes, with this increased eating time potentially coming at the expense of lying time, which was approximately 0.5 to 1 h less than for the 7RG and HFRG treatments, respectively. The CG farmlet had the lowest APC for the majority of the study (Figure 1), which likely contributed to the increased time spent eating in the CG cows as grazing time increases at lower sward surface heights to compensate for lower bite mass and rates (Gibb et al., 1999). Average lying bout duration appeared to be similar between treatments; thus, the lower lying time for cows in the CG treatment appears to be driven by fewer lying bouts (Table 1). Average lying times of healthy lactating dairy cows on pasture in New Zealand have recently been reported to range between 7.6 and 10.1 h/d (overall average of 8.7 h/d; Schutz et al., 2020), and the average lying times of cows in the HFRG and 7RG groups were within this range. However, the daily lying time for cows in the CG treatment averaged less than the 8 h recommended for grazing dairy cows (DairyNZ, 2023), suggesting the treatment effects on APC resulted in potentially compromised lying time and cow welfare (Munksgaard and Simonsen, 1996).

**Table 1:** Average milk production, animal characteristics and behavior, and pasture estimates over the 12-wk experimental period, for high frequency rotational grazing (HFRG), rotational grazing with weekly allocations (7RG), and continuous grazing (CG)^1^

Parameter	HFRG	7RG	CG	Maximum SED	*P*-value^2^
Milk production					
Milk weight (kg/cow per day)	24.3^b^	25.2^a^	25.0^ab^	1.34	<0.05
Fat yield (kg/cow per day)	1.11	1.13	1.13	0.066	0.66
Protein yield (kg/cow per day)	0.89^b^	0.92^a^	0.95^ab^	0.048	<0.05
Lactose yield (kg/cow per day)	1.17^b^	1.22^a^	1.20^ab^	0.066	<0.05
Milk solids yield (kg/cow per day)	2.00	2.05	2.08	0.114	0.28
FPCM yield^3^ (kg/cow per day)	27.1	27.9	27.8	1.56	0.30
Animal characteristics and behavior					
Liveweight (kg/cow)	502	510	481	17.5	0.11
BCS	4.3	4.3	4.2	0.24	0.65
Eating time (min/d)	495^b^	525^b^	623^a^	13.7	<0.01
Ruminating time (min/d)	403^a^	420^a^	338^b^	18.3	<0.01
Active time (min/d)	131	128	151	14.3	0.22
High active time (min/d)	136^a^	138^a^	102^b^	7.0	<0.01
Not active time (min/d)	269^a^	232^b^	231^b^	9.5	<0.01
Steps (n/d)	3,823^b^	3,751^b^	4,440^a^	119.0	<0.01
Lying bouts (n/d)	7.5^a^	7.4^a^	6.6^b^	0.26	<0.01
Lying time (min/d)	497^a^	475^a^	440^b^	13.7	<0.01
Pasture estimates					
Energy required (MJ of ME/cow per day)	206	209	213	12.6	0.72
Pasture required^4^ (kg of DM/cow per day)	17.9	18.0	18.6	1.10	0.82
Pasture required (kg of DM/ha)	4,134	4,160	4,285	—	—
Baleage harvested (kg of DM/ha)	1,140	767	0	—	—
Change in pasture mass^5^ (kg of DM/ha)	404	8	234	—	—
Apparent pasture growth (kg of DM/ha per day)	68	59	54	—	—
Average pregrazing height (mm)	96	86	—	6.2	0.12
Average postgrazing height (mm)	56	51	—	3.7	0.20

a,b Mean values within a row with the same superscript are not significantly different at *P* < 0.05, by Tukey's test.

1 Values are least squares means when *P*-values are provided. One cow in the CG herd was removed in wk 6 to maintain sufficient feed availability; a zero value for milk, liveweight, and BCS was assigned to ensure averages were comparable across farmlets. SED = standard difference of means.

2 *P*-value for the pretrial covariate (except grazing height) and treatment × week interaction (except BCS) was *P* < 0.05 for all parameters.

3 FPCM = fat- and protein-corrected milk yield.

4 Accounting for the 1 bale of pasture baleage (180 kg of DM) offered to the 7RG herd.

5 Change from start to finish of the 12-wk experiment.

**Figure 1:**
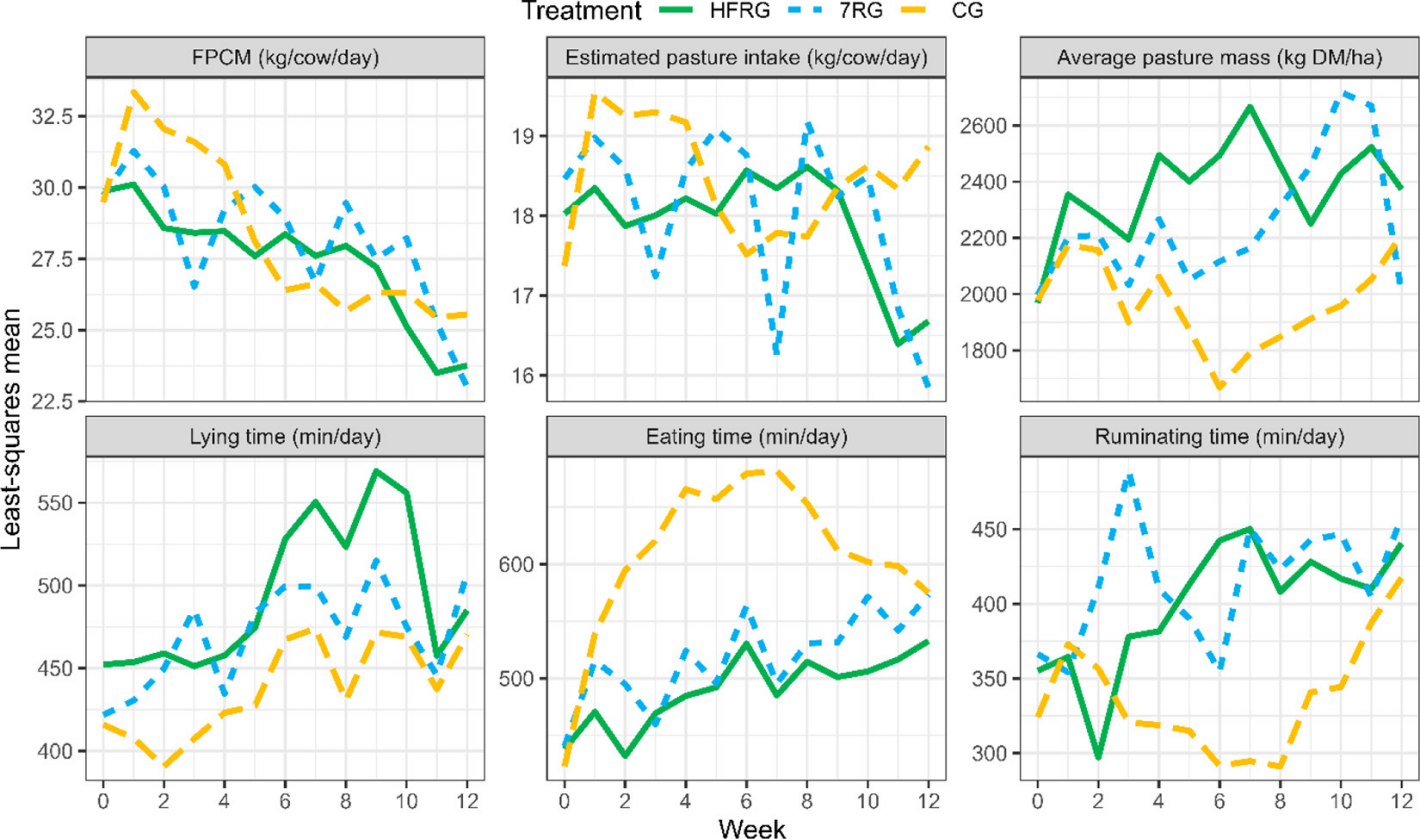
Fat- and protein-corrected milk yield (FPCM), back-calculated estimated pasture intake, pasture cover, and lying, eating, and ruminating time (min/d) by treatment and week, for high frequency rotational grazing (HFRG), rotational grazing with weekly allocations (7RG), and continuous grazing (CG).

Within the week, the HFRG and CG treatments had relatively consistent daily lying and eating times compared with the 7RG treatment (Figure 2). At the beginning of the week when feed was abundant the behavior of cows in the 7RG treatment appeared more similar to HFRG. Toward the end of the week as sward surface height decreased the behavior of cows in the 7RG treatment appeared to be more similar to those in the CG treatment. Corresponding changes in milk production can also be seen in Figure 2, where at the start of the 7-d period (Friday) the 7RG cows were essentially offered ad libitum pasture. Toward the end of the week, production decreased as feed availability declined, and production recovered over several days once cows received their new allocation. The peak in milk production at d 4 of the 7RG pasture allocation frequency was similar to previous studies with multiday residence time in an allocation (McMeekan, 1956; Hoden et al., 1991).

**Figure 2:**
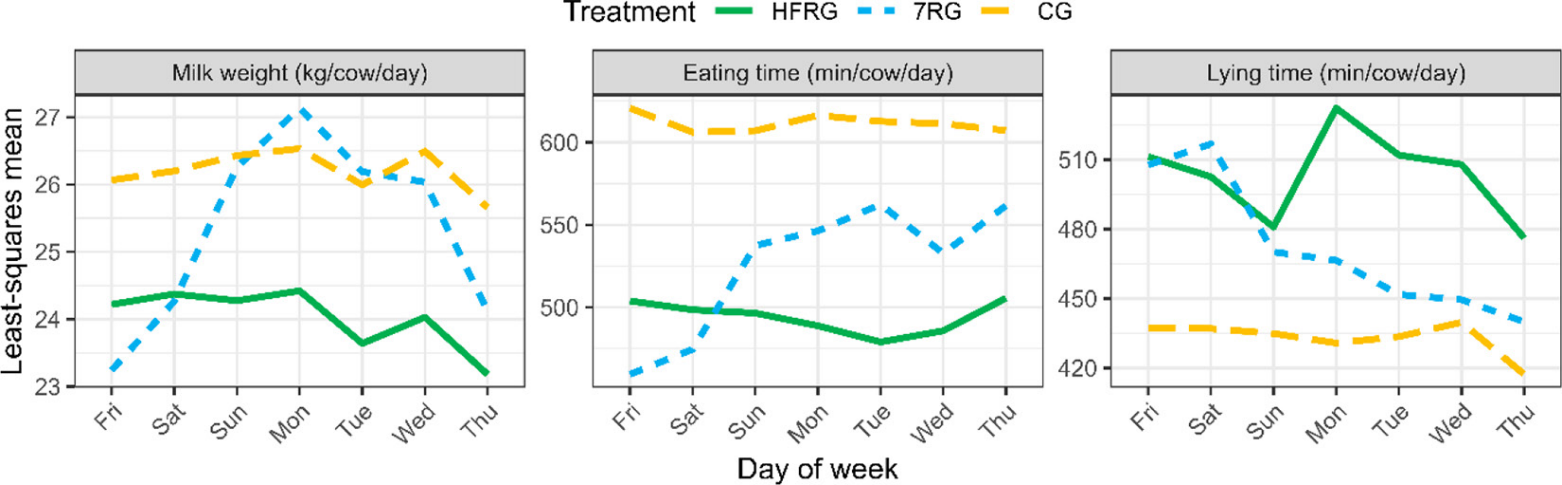
Milk weight, eating time, and lying time by day of the week, for high frequency rotational grazing (HFRG), rotational grazing with weekly allocations (7RG), and continuous grazing (CG). Effect of treatment and treatment × day of week interaction was significant at *P* < 0.05, with the exception of the treatment effect on milk weight, which was not significant (standard difference of means = 1.86).

The change in BCS from start to end of the experimental period was not different between groups. Consequently, BCS averages are reported in Table 1. Milk production was initially greatest for CG cows as they effectively had ad libitum pasture, declining as pasture availability (indicated by APC) decreased (Figure 1). Lack of treatment effects on FPCM production (Table 1) are supported by previous studies comparing daily pasture allocations with each of the frequencies explored in this study (McMeekan, 1956; Dalley et al., 2001; Pulido and Leaver, 2003). Our results reaffirm a lack of effect on overall FPCM production from even relatively extreme differences in pasture allocation frequency. Estimated pasture growth was highest in the HFRG treatment and lowest for CG, with 7RG having an intermediate value. Lower pasture yield with lower frequency of pasture allocation was expected (McMeekan, 1956; Lucas, 1958) and has implications for optimum stocking rates and farm profitability. This serves as a reminder that rotational grazing is beneficial for pasture production rather than increased animal intake and milk production. The size of the trade-off in pasture yield relative to the value of reduced work and cognitive load from fewer grazing management decisions needs to be determined, including investigating allocation frequencies of >1 and <7 d. It is unclear from our study what effect changing stocking rate alongside pasture allocation frequency would have on animal behavior.

Our results confirm the ability of dairy cows to differ in their behavior to meet their daily feed demand and differences in FPCM production were not statistically significant or biologically important when responding to wide variations in pasture allocation frequency and resulting changes in pasture sward characteristics. Should the trade-off between workload, cognitive load, and pasture production be favorable, farmers could reduce allocation frequency, for at least a period, without compromising eating, ruminating, and lying time. However, caution should be taken with continuous grazing where cows spent more time eating and their daily lying time appeared to be compromised.
